# Thoracic oncology multidisciplinary teams: Between the promises and challenges

**DOI:** 10.4103/1817-1737.38395

**Published:** 2008

**Authors:** Abdul-Rahman Jazieh, Abdulrahman Al Hadab, John Howington

**Affiliations:** Department of Oncology, King Abdul-Aziz Medical City, King Fahad National Guard Hospital (KAMC-KFNGH), Saudi Arabia; * *Northwestern University Feinberg School of Medicine, Evanston Northwestern Healthcare, Department of Surgery, Evanston, IL, USA*

**Keywords:** Multidisciplinary teams, thoracic oncology

## Abstract

The thoracic oncology multidisciplinary teams are playing an increasing role in the management of thoracic malignancies. These teams have a great potential to improve the patient care and the health care system, however, they are faced by many challenges. To realize the full potential of these teams, a better understanding of their functions, roles, benefits and challenges from all involved including teams members and leadership is crucial.

## Introduction

Multidisciplinary team (MDT) is a frequently repeated term in the healthcare arena, which denotes a healthcare group with multiple members from different backgrounds and expertise providing complementary services translated into efficient and comprehensive care. The composition and functions of these teams may vary dependent on the settings and the organizational environment. In the present manuscript, we will discuss the thoracic oncology MDT including justification, definition, functions and challenges.

## Why a Multidisciplinary Approach?

The multidisciplinary approach has become very crucial recently due to the ever-increasing complexity of medical knowledge and the huge wealth of information that is available to physicians, in addition to the complexity of the various medical procedures and interventions available for cancer care. Furthermore, the development of subspecialization in very narrow medical disciplines has made specialist expertise and input more valuable. The MDT plays a critical role in the whole spectrum of cancer management including diagnosis, staging, treatment and palliative care.[1,2]

The approach to an individual patient is usually dependent on the presumptive diagnosis; and obtaining a correct diagnosis involves weighing the risk factors, symptoms, laboratory findings and radiographic features. The confirmation of a presumptive diagnosis may involve several subspecialties including chest radiologist, interventional radiologist, pulmonary physician, gastroenterology endoscopist, thoracic surgeon and pathologist. Putting the pieces of the puzzle of the clinical scenario together requires significant and frequent interactions among the team members to reach the right conclusion.

Staging lung cancer often requires mediastinal lymph node biopsy, which could be done by pulmonary physicians through Wang's needle biopsy, a gastroenterologist with endoscopic ultrasound expertise or by thoracic surgeons through mediastinoscopy. The decision should be based on the location of the tumor, target lymph nodes and the expertise available. This decision requires input from the different team members.

The optimal treatment also requires various subspecialty expertise and ranges from local endobronchial therapy (brachytherapy) to open the airway, to external beam radiotherapy, to radiosurgery, to surgical resection and systemic therapy or combined modality treatment. Treatment plans require significant coordination and communication between the different team members. The existence of an MDT enhances the evidence-based approach to cancer care because physicians have to justify their decisions and actions when a case is put forward for the discussion in a group format. This critical review among experts from various disciplines enhances the compliance with evidence-based medicine guidelines.

Palliative care for lung cancer includes relieving post-obstruction symptoms, pleural effusion management, pain management and superior vena cava obstruction management, etc. Addressing these problems requires interventions from different specialists as well.

## What is Multidisciplinary Care?

The old approach of multidisciplinary care was to send the patient to individual specialists at various locations with often extended time intervals between the appointments. Communication between these specialists was by phone, e-mail or regular mail. Face-to-face discussion rarely took place except when physicians ran into each other accidentally and, at that time, it was difficult to discuss the case in a very systematic way. The optimal multidisciplinary care setting is a system that allows the concurrent exchange of input from different disciplines and for formal presentation of each case with all needed information available at the time of discussion including the diagnostic work-up. Consensus judgment about available data and about a particular patients′ issue will be made during the MDT meeting. Of course, devising the treatment plan should be evidence-based.

There are misconceptions about multidisciplinary care among some physicians. One misconception and concern is the loss of control of patients referred to a doctor in the MDT. Another misconception is that the patient will be seen by all physicians in the team whether they are needed to be involved or not, which is viewed correctly as an inefficient way of using specialists′ time and would add unnecessary costs.

**Figure 1:**
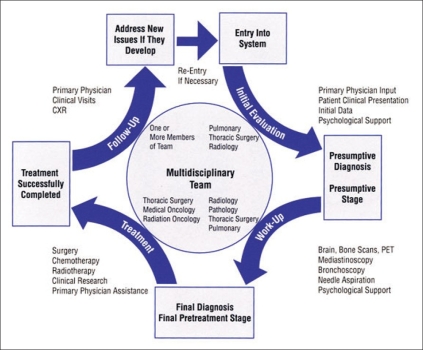
The flow process in a thoracic multidisciplinary team care

Figure 1 shows the flow system in the multidisciplinary thoracic team.

## Team Members

The team members of a multidisciplinary thoracic oncology program include pulmonary medicine specialists, chest radiologists, thoracic surgeons, medical oncologists, radiation oncologists, lung pathologists, GI medicine specialists and other disciplines such as nurses, tumor registrars, research coordinators, etc.

## The MDT Conference (tumor board)

It is crucial that the MDT holds at least one weekly conference where all new patients and challenging cases are discussed. The participation of all the team members, especially radiologists and pathologists, is crucial. The team should make a collective judgment to where the particular aspect of each situation is discussed thoroughly. The team will discuss the diagnostic procedures, staging work-up and the sequence in which tests or interventions are pursued. The team members can carry out a collective interpretation of existing data regarding treatment outcomes and discuss tentative treatment plans. To keep the conference relevant and viable, the team members must be kept engaged and decisions for treatment must be based on evidence-based data, not on unfounded personal opinions. When there is paucity of evidence or conflicting evidence about the best approach to care, the opinion of each participating specialist should be welcomed and respected. A significant advantage to the MDT approach is the discussion and consensus occurs among the specialists away from the patient, and a unified approach is presented to the patient, which avoids the confusion of hearing disparate opinions among specialists as the patient travels from office to office.

With the MDT approach, there will be collective and joined discussion and decisions about the best diagnostic procedure to perform as well as the most accurate clinical staging procedures. Finally, the team has the ability to immediately devise the best multidisciplinary treatment for the patient when appropriate. This will have many advantages to the physician who will get immediate help in working up the patients and in making treatment decisions, making patient care more efficient and more satisfying. It will improve the communication between the various physicians involved in thoracic oncology care and help organize the thoracic oncology program overall. There will be multiple advantages to the patient including seeing multiple specialists in one visit if needed and receiving a consistent message without confusion due to the physical proximity between the specialists and face-to-face discussion among specialists. Therefore, the patient will have one stop shopping: evaluation, further testing and a consensus plan.

## Type of Multidisciplinary Clinics

There are physical clinics where several specialties share clinic space. The patient may come to see one or more specialists and not necessarily all the specialists on the same day. On the other hand, there is a virtual clinic where a patient is seen by specialists at separate locations, but at a defined weekly meeting all disciplines meet and discuss the case together.

## Development of Multidisciplinary Thoracic Oncology Team

There are a few crucial components of any MDT including the personnel, the conference, the clinic, the marketing and the patients.

### Personnel

There should be critical mass of participation by different specialties mentioned above. Preferably, there should be two specialists in each discipline to enable an unbiased discussion and presenting different opinions in difficult cases. This also allows for input from each discipline when an individual specialist is away or unable to attend the weekly conference. Table 1 depicts the specialists and duties of a thoracic oncology MDT. There should be, of course, administrative staffing to support the team at different levels such as correspondence, minutes and organizing the activities as well as a clinical coordinator to follow up on the team recommendations such as appointments and test scheduling. The personnel should have the right attitude to make this successful. They should be committed to work together and have mutual respect for the expertise of each discipline and therefore, arguments should not be based on personal opinions but based on data and evidence. There should be respect for different opinions and the team should on occasion agree to disagree in order to have a rich environment for growth and input.

**Table 1:** Depicts the specialists and their roles and expertise in the thoracic oncology multidisciplinary team

Disciplines	Roles and expertise			
	
	Diagnosis	Staging	Treatment	Palliative care
Thoracic surgery	Obtain tissue by thoracotomy, mediastinoscopy	Mediastinoscopy	Surgical resection	Surgery, pleurodesis
Pulmonary	Obtain tissue by bronchoscopy	Work-up + wang's needle biopsy	-	Endobronchial treatment, pleurodesis stenting
Medical oncology	-	Work-up	Systemic therapy	Symptom control
Radiation oncology	-	Work-up	Radical therapy	CNS radiotherapy, pain, superior vena cava syndrome, cord compression
Pathology	Pathological diagnosis	-	-	-
Radiology[3]	Imaging studies	Imaging studies	Tumor response evaluvation	Interventional radiology, SVC stents
Gl endoscopist	Tissue by Endoscopic Ultrasound	Biopsy of mediastinal lymph node	-	Stenting esophagus

### Conference

There should be a weekly meeting in a conference format that is well organized in advance to have all the necessary data, images and slides required in order to make relevant real time discussions as well as treatment decisions. Of course, there is a need to have an adequate number of patients to discuss in order to make the best use of the participants time with an estimate of 150 patients/year.

### Marketing

The concept will market itself as most referring physicians will be happy to send their patients to a one-stop shop. It will not only be convenient but also they will have more value and trust in MDT.

## Functions of MDT

### Providing the best patient care

The best care is state-of-the-art care, which is efficient and comprehensive. MDTs should be capable of providing this more effectively than any individual provider, as the team will be able to complete the work-up and devise a management plan more expeditiously.

### Improving the healthcare system delivery

The team will be able to detect any systemic error such as a delay in acting upon abnormal chest X-rays. If the team discovers a delay in diagnosis of a lung mass, they can evaluate any systemic error and fix it. This will also create a reliable referral network for internal and external physicians. The team could also develop clinical guidelines and implement them for the thoracic malignancies. This in turn will improve the healthcare system leadership's confidence and support to the team and enable the multidisciplinary thoracic oncology team to further improve the system. In addition, patient's confidence and satisfaction will improve significantly when they realize that there are multiple specialists focused on their condition.

### Education

The team is the best medium to educate the team members as well as other colleagues in the institution. The multidisciplinary conference is an excellent educational opportunity for residents and students. The team could conduct educational activities for healthcare professionals at the national or international level in addition to public outreach educational activity in the field of thoracic oncology.

### Research

The team members can identify areas of interest and needs and then discuss and evaluate the feasibility and justification of studies, which enable them to develop collaborative projects and improve accrual to studies.

### Career development and job satisfaction

The physicians in the team will have the sense of belonging to a professional group. The team may offer practical mentoring from the senior physicians to the juniors and improve the education and research experience of everyone. Furthermore, this will help the new physicians to have access to expertise in the organization in a very systematic way.

## Challenges of MDT

These challenges can be categorized into three categories: lack of institutional support and infrastructure, lack of motivation and lack of time.

### Lack of institutional support and infrastructure

The MDT needs a space for conferences and a clinic and support staff for centralized coordination. The physicians need a protected time and backup to be able to do these functions. Therefore, the MDT function is an institutional activity and not just an individual physician activity. It should be adapted at a departmental level or higher to be made successful.

### Lack of motivation

Some physicians may not see the value of the team; therefore, there should be a benefit to participation such as intellectual stimulation, continuing medical education, etc. This activity should be endorsed by the leadership to encourage physician participation and help in making the resources accessible for the team members.

### Lack of time

The MDT approach can save time, such as not having to call multiple specialists′ review pathology and radiology at different times and locations or trying to track one another down to discuss management. This will enable a physician to have a one-stop shop for all these activities. The support system should minimize physician burden and arrange physician backup during the multidisciplinary meeting. The most important issue of the team is to be predictable and consistent and be at a time that is most convenient to all team members.

## Conclusion

Thoracic oncology MDTs are critical for patient care, career development and improving healthcare delivery.

Institutions that take upon themselves providing care to patients with thoracic malignancies should encourage the establishment of these teams and provide the necessary support for their success.
